# Myc Is Required for Activation of the ATM-Dependent Checkpoints in Response to DNA Damage

**DOI:** 10.1371/journal.pone.0008924

**Published:** 2010-01-27

**Authors:** Lina Guerra, Ami Albihn, Susanna Tronnersjö, Qinzi Yan, Riccardo Guidi, Bo Stenerlöw, Torsten Sterzenbach, Christine Josenhans, James G. Fox, David B. Schauer, Monica Thelestam, Lars-Gunnar Larsson, Marie Henriksson, Teresa Frisan

**Affiliations:** 1 Departments of Cell and Molecular Biology, Karolinska Institutet, Stockholm, Sweden; 2 Departments of Microbiology, Tumor and Cell Biology, Karolinska Institutet, Stockholm, Sweden; 3 Division of Biomedical Radiation Sciences, Rudbeck Laboratory, Uppsala University, Uppsala, Sweden; 4 Institute for Medical Microbiology and Hospital Epidemiology, Hannover Medical School, Hannover, Germany; 5 Department of Biological Engineering, Division of Comparative Medicine, Massachusetts Institute of Technology, Cambridge, Massachusetts, United States of America; 6 Department of Plant Biology and Forest Genetics, Swedish University of Agricultural Sciences, Uppsala, Sweden; Roswell Park Cancer Institute, United States of America

## Abstract

**Background:**

The MYC protein controls cellular functions such as differentiation, proliferation, and apoptosis. In response to genotoxic agents, cells overexpressing MYC undergo apoptosis. However, the MYC-regulated effectors acting upstream of the mitochondrial apoptotic pathway are still unknown.

**Principal Findings:**

In this study, we demonstrate that expression of Myc is required to activate the Ataxia telangiectasia mutated (ATM)-dependent DNA damage checkpoint responses in rat cell lines exposed to ionizing radiation (IR) or the bacterial cytolethal distending toxin (CDT). Phosphorylation of the ATM kinase and its downstream effectors, such as histone H2AX, were impaired in the *myc* null cell line HO15.19, compared to the *myc* positive TGR-1 and HOmyc3 cells. Nuclear foci formation of the Nijmegen Breakage Syndrome (Nbs) 1 protein, essential for efficient ATM activation, was also reduced in absence of *myc*. Knock down of the endogenous levels of MYC by siRNA in the human cell line HCT116 resulted in decreased ATM and CHK2 phosphorylation in response to irradiation. Conversely, cell death induced by UV irradiation, known to activate the ATR-dependent checkpoint, was similar in all the cell lines, independently of the *myc* status.

**Conclusion:**

These data demonstrate that MYC contributes to the activation of the ATM-dependent checkpoint responses, leading to cell death in response to specific genotoxic stimuli.

## Introduction

The MYC transcription factor regulates a wide variety of cellular functions such as proliferation, differentiation, cellular motility, and apoptosis (reviewed in [1]). Since it controls the delicate balance between cell proliferation and cell death, MYC expression can be found deregulated in many different types of tumors [2], [3], [4]. The oncogenic capacity of MYC is not only dependent on its ability to regulate expression of genes that enhance cell proliferation (e.g up-regulation of cyclin D2, Cdk4 and E2F) or suppress cell cycle arrest (e.g. down-regulation of p21^CIP1^ or p15^INK4B^, reviewed in [5]), but it may contribute to tumorigenesis by induction of DNA damage, and consequent genomic instability [6], [7], [8], [9].

Stress signals, such as growth factor deprivation, hypoxia, and exposure to genotoxic agents trigger MYC dependent apoptosis [10], [11]. The apoptotic response is dependent on release of cytochrome C from the mitochondria through activation or up-regulation of the pro-apoptotic members of the Bcl-2 family [10], [12], [13], [14]. Our previous studies were aimed at understanding the role of MYC in cells exposed to conventional DNA damaging cytotoxic drugs. We showed that ectopic expression of MYC sensitized cells to apoptosis induced by etoposide and camptothecin in cancer cells [15], [16], [17]. Similarly, doxorubicin, camptothecin and etoposide induced a strong apoptotic response in the Rat1 cell line TGR-1, expressing physiological levels of Myc, via activation of Bid and Bax. Cell death in response to camptothecin and etoposide was further enhanced by the pro-apototic enzyme PKCδ [16], but inhibited by the heat shock protein 70 [17]. The apoptotic response was dependent on Myc expression, since activation of these proteins was not detected in the *myc* null cells HO15.19 [15], [16]. However, the molecular signaling pathway that regulates the Myc dependent Bax activation in response to DNA damage is not known.

In normal cells, exposure to genotoxic agents triggers activation of checkpoint responses that lead to cell cycle arrest or cell death. A key protein in orchestrating the complex response to DNA double strand breaks (DSB) is the Ataxia telangiectasia mutated (ATM) kinase [18]. ATM exists as an inactive dimer, which undergoes a conformational change upon induction of DNA DSB. The change in protein structure stimulates intermolecular autophosphorylation on Ser1981, resulting in dissociation of the dimer, and consequent activation of the kinase [19]. Full activation of ATM requires interaction with the MRN complex (MRE11/RAD50/NBS1), which enhances the recruitment of ATM to the site of DNA damage [20], [21]. Proteins identified as ATM substrates regulate recruitment of DNA repair complexes (e.g. H2AX), activate checkpoint responses to block cell proliferation (e.g. CHK2 kinase), or induce apoptosis (e.g. the tumor suppressor p53) [22]. Despite the well-established role of MYC in activating p53-dependent apoptosis, the exact role of MYC in regulating the DNA damage response remains poorly understood [23], [24], [25], [26].

To identify the MYC-regulated effectors acting upstream of the mitochondrial apoptotic pathway in response to genotoxic stress, we have used Rat1 cells with different *myc* status: the parental TGR-1 cells expressing physiological levels of Myc, the *myc* null cells HO15.19, and the HOmyc3 cells, where expression of the murine *myc* has been reconstituted [27], [28]. We demonstrate that *myc* deletion impairs activation of the ATM dependent DNA damage checkpoint responses in cells exposed to ionizing radiation (IR) or the cytolethal distending toxin (CDT), including impaired phosphorylation of ATM and its downstream target H2AX, and reduced nuclear foci formation of the MRN complex. The role of MYC in the regulation of the ATM-dependent responses to genotoxin agents was further confirmed in the HCT116 cell line, where the endogenous levels of MYC were knocked down by specific siRNA.

These data contribute to the understanding of the function of MYC as a regulator of the ATM-dependent checkpoint responses in response to irradiation or intoxication with CDT.

## Materials and Methods

### Cell Lines

*Myc* null HO15.19 Rat1 fibroblasts were generated from parental TGR-1 cells by homologous recombination, deleting both *myc* alleles, and HOmyc3 cells were generated by reconstitution of the murine *myc* gene into the HO15.19 cells [27], [28]. The HCT116 cell line [29] was obtained from ATCC. Cells were cultivated in Dulbecco's modified eagle medium (DMEM), supplemented with 10% fetal calf serum (FCS) and 5 mM L-glutamine (complete medium) in a humid atmosphere containing 5% CO_2_.

### Treatments

#### CDT intoxication

Cells were incubated for the indicated time periods with bacterial lysate (diluted 1∶2000 in complete medium) derived from the *Helicobacter hepaticus* strain ATCC51449, expressing the wild type CDT, or the control strain *H. hepaticus cdtB*, (HhcdtBm7) expressing a mutant inactive CDT [30]. Bacteria were cultured on a rotary shaker to mid-logarithmic growth phase in liquid medium (Brucella broth, supplemented with 10% horse serum) and in a microaerobic atmosphere (10% hydrogen, 10% CO_2_, 80% nitrogen). Bacteria were then centrifuged from the liquid medium, and the pellet washed twice with PBS, and sonicated for 5 min in a Branson sonicator. The lysates were filtered-sterilized and stored at −20°C. The lysate dilution of 1∶2000 represents the minimal dose sufficient to induce cell cycle arrest in 100% of the cell population.

#### Ionizing radiation

Cells were irradiated (20Gy), washed once with PBS and incubated for the indicated periods of time in complete medium.

#### 

*UV irradiation.* Cells were irradiated for 3 min with a short wave UV lamp (model UVG-11, 254 nm, 220 V, 0.12 AMPS, 50 Hz, UVP, Upland, USA), washed once with PBS and incubated in complete medium for the indicated periods of time.

### Cell Cycle Analysis

Cells were trypsinized, centrifuged and washed once with PBS. The cell pellet was resuspended and fixed overnight with 1 ml cold 70% ethanol at 4°C. The cells were subsequently centrifuged and resuspended in 0.1 ml propidium iodide (PI) solution (0.05 mg ml^−1^ PI and 0.25 mg ml^−1^ RNase in PBS) for 30 min at 4°C. Flow cytometry analysis was performed using a FACScan cytometer (Becton Dickinson). Data from 10^4^ cells were collected and analyzed using the CellQuest software (Becton Dickinson).

### Immunofluorescence

Fifty thousand cells/well, plated on round 13 mm glass coverslips in 24-well plates in complete medium, were left untreated, intoxicated or irradiated, and incubated for the indicated periods of time. Cells were fixed with 4% paraformaldehyde, and permeabilized with 0.5% Triton X-100 for 30 min at RT. Slides were blocked in PBS with 3% BSA for 30 min and incubated for 1h at RT with a α-NBS1 mAb antibody (BD Transduction Laboratories) diluted 1∶50 in PBS. Slides were washed three times for 5 min in PBS, and then incubated with fluorescein-conjugated (FITC)-conjugated anti-mouse antibody (DAKO), diluted 1∶100 in PBS for 30 min at RT. Nuclei were counterstained with Hoechst 33258 (Sigma, 0.5 µg ml^−1^) for 1 min at RT. Slides were mounted and viewed by fluorescence microscopy with a Leica DMRXA fluorescence microscope with a CCD camera (Hammamatsu) and images were captured with the Improvision Openlab v.2 software.

### Western Blot Analysis

Proteins were separated in SDS-polyacrylamide gels, transferred to PVDF membranes (Millipore) and probed with the relevant antibodies. The following antibodies were used: α-phospho-H2AX (Upstate Biotechnology), α-phospho-ATM (Ser1981, Novus Biologicals), α-ATM (Genway), α-p53 and α-MYC (9E10) (Santa Cruz Biotechnology, Inc.), α-actin (Sigma), α-phospho-p53 (Ser15) and α-phospho-CHK2 (Thr68) (Cell Signaling), and α-NBS1 (BD Transduction Laboratories). Blots were developed with enhanced chemiluminescence using the appropriate horseradish peroxidase-labeled secondary antibody, according to the instructions from the manufacturer (GE Healthcare).

### siRNA

HCT116 cells were transfected with double stranded siRNA targeting: Myc: (5′-CGAGCUAAAACGGAGCUUUdTdT-3′), or GFP as negative control (5′-GGCUACGUCCAGGAGCGCAdTdT) at a final concentration of 50nM using Lipofectamin 2000 (Invitrogen), and incubated in complete medium for 48h.

### Detection of Endogenous MYC Levels

For analysis of MYC expression, cells were harvested in lysis buffer (1mM PMSF, 100mM Tris pH8, 150mM NaCl, 5mM EDTA, 1% NP-40, 0.5% Trasylol, Complete Protease Inhibitor), and the MYC protein was immunoprecipitated using an α-MYC-specific antibody (N262, Santa Cruz). Proteins were separated in 10% SDS-polyacrylamide gels, transferred to PVDF membranes (Millipore) and probed with the α-MYC specific antibody C-33 (Santa Cruz).

## Results

**Figure 1:**
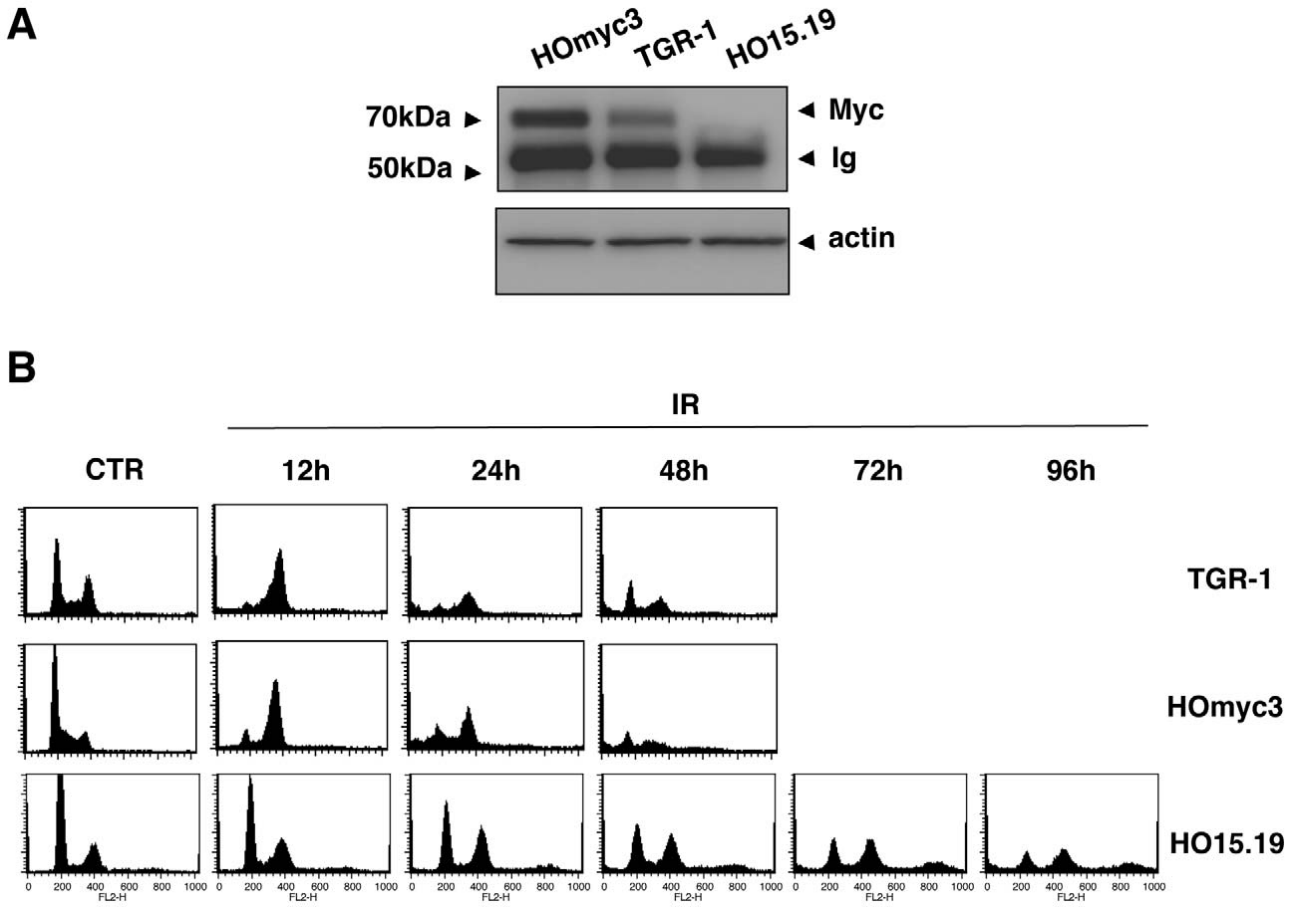
*myc* deletion delays cell death upon irradiation. **A**) The levels of the endogenous Myc protein were assessed by immunoprecipitation followed by western blot analysis in TGR-1, the *myc* reconstituted cells HOmyc3, and the *myc* null HO15.19 cells using α-Myc antibodies. Expression of actin in total cell lysates was used as control. **B**) TGR-1, the *myc* reconstituted cells HOmyc3, and the *myc* null HO15.19 cells were left untreated or irradiated (20Gy) and further incubated in complete medium for the indicated periods of time. Analysis of the cell cycle distribution was assessed by PI staining and flow cytometry as described in Material and Methods. One out of four independent experiments is shown.

Myc enhances the apoptotic effect of DNA damaging agents, such as etoposide and camptothecin, by activation of the pro-apoptotic protein Bax, and the downstream caspases 3 and 9 [15], [16], [17]. To identify the Myc-regulated effectors acting upstream of the mitochondrial apoptotic pathway, we used the Rat1 fibroblasts TGR-1 expressing physiological levels of Myc, the isogenic *myc* null HO15.19 cell line, and the HOmyc3 cell line, where expression of the murine *myc* gene was reconstituted into the HO15.19 background [27]. The levels of expression of the endogenous Myc were assessed by immunoprecipitation followed by western blot analysis using an α-Myc-specific antibody. As shown in Figure 1A, the HOmyc3 cell line presented approximately a 3-fold increase in Myc expression compared to the parental TGR-1 cells. As expected, Myc was not detected in the HO15.19 cells.

**Figure 2:**
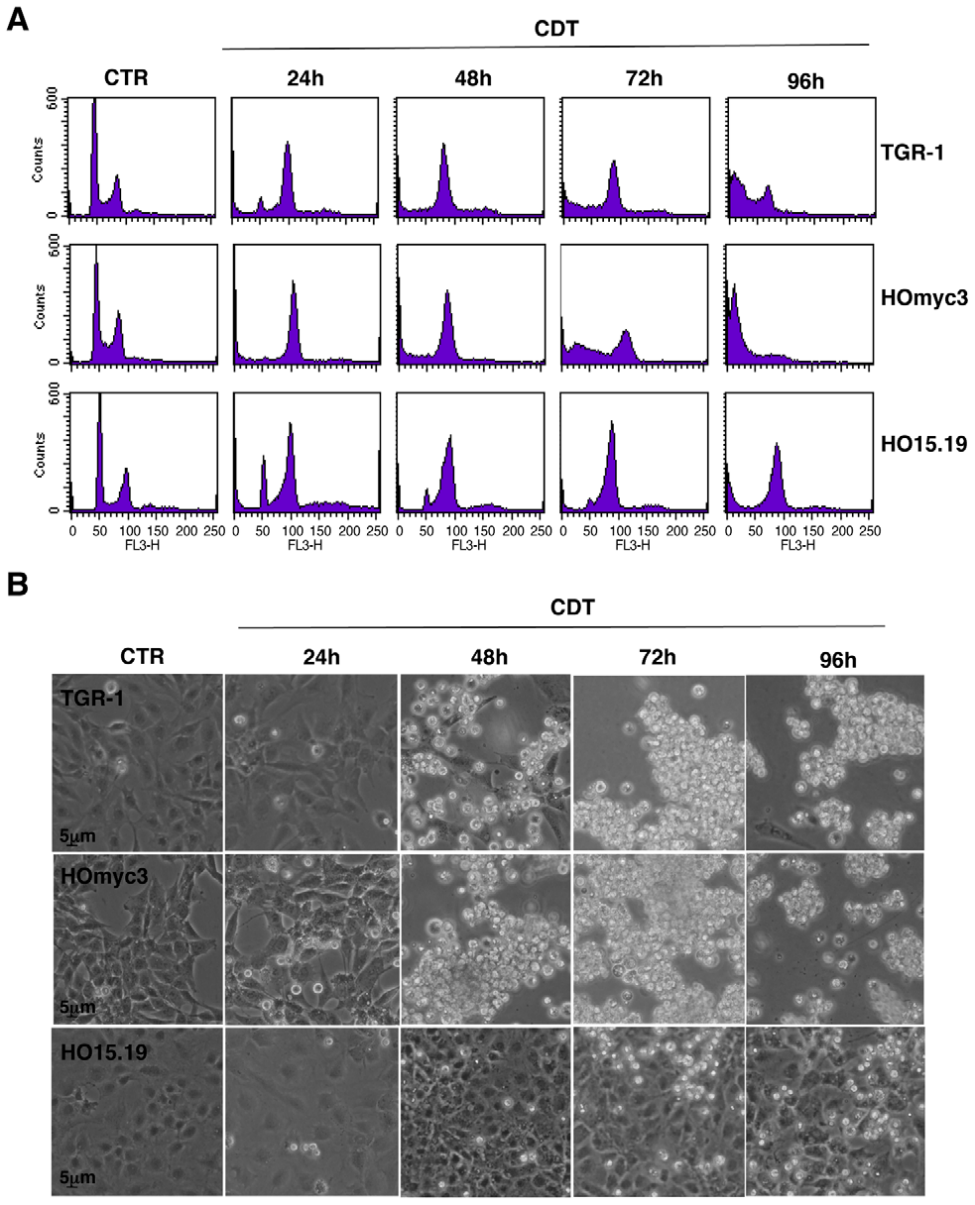
*myc* deletion delays cell death upon CDT intoxication. TGR-1, the *myc* reconstituted cells HOmyc3, and the *myc* null HO15.19 cells were exposed to bacterial lysates (1∶2000) expressing the mutant (CTR) or the wild type form (CDT) of *H. hepaticus* CDT and further incubated in complete medium for the indicated periods of time. **A**) Analysis of the cell cycle distribution assessed by PI staining and flow cytometry as described in Material and Methods. **B**) Phase contrast micrographs of the cells taken at the indicated time points (Magnification 40×). One out of three independent experiments is shown.

**Figure 3 pone-0008924-g003:**
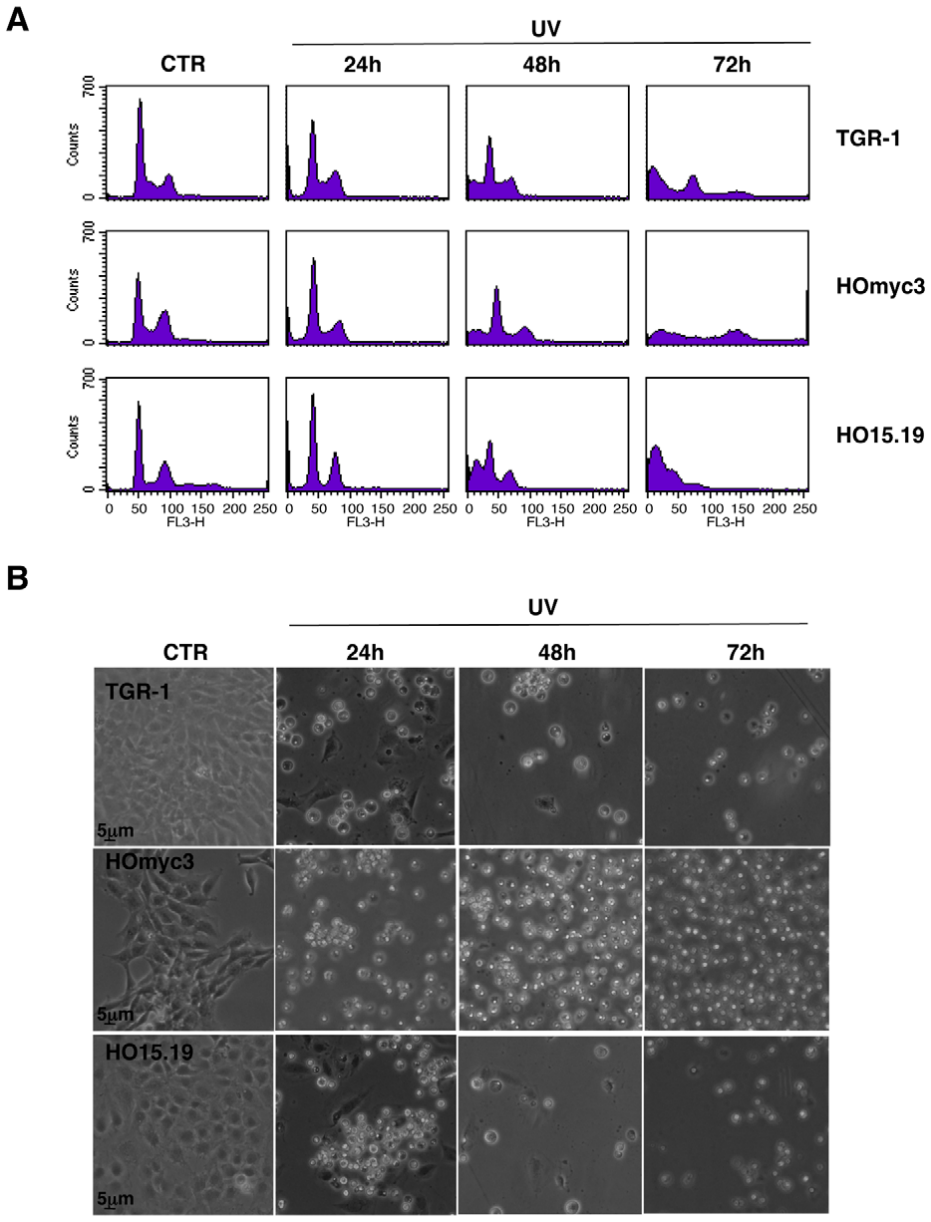
*myc* deletion does not alter cell death kinetics upon UV irradiation. TGR-1, the *myc* reconstituted cells HOmyc3, and the *myc* null HO15.19 cells were left untreated or exposed to UV irradiation and further incubated in complete medium for the indicated periods of time. **A**) Analysis of the cell cycle distribution assessed by PI staining and flow cytometry as described in Material and Methods. **B**) Phase contrast micrographs of the cells taken at the indicated time points (Magnification 40×). One out of three independent experiments is shown.

As DNA damaging agents, we chose ionizing radiation (IR) and the bacterial cytolethal distendin toxin (CDT), known to cause DNA double strand breaks (DSBs) and activate the ATM-dependent checkpoint responses [22], [31], [32]. Upon irradiation, the Myc expressing TGR-1 and HOmyc3 cells were arrested in the G2 phase of the cell cycle 12h after treatment. Arrest in G2 was followed by cell death, assessed as increase of the sub-G1 cell population with fragmented DNA, 24h and 48h after irradiation (Figure 1B). A delayed arrest in G2 was observed in the *myc* null cells HO15.19 24h to 48h after treatment, and signs of cell death occurred only 72h–96h post-irradiation (Figure 1B). Similarly, the *Helicobacter hepaticus* CDT induced G2 arrest both in the TGR-1 and HOmyc3 cells, followed by cell death, as assessed by an increase of the sub-G1 population 72h and 96h after intoxication (Figure 2A). CDT intoxication of the HO15.19 cells led to delayed G2 arrest that was completed at 72h, and minimal signs of cell death were observed as late as 96h after treatment (Figure 2A). These data were confirmed by analysis of the cell morphology. A massive cell detachment was observed in intoxicated TGR-1 and HOmyc3 cells exposed to CDT for 48h, while this effect was not as pronounced in the HO15.19 cell line even at 96h post-intoxication (Figure 2B). UV irradiation, which causes dipyrimidine photoproducts and activates mainly the ATR-dependent DNA damage response [31], induced cell death with similar kinetics in all the cell lines tested, independently of the *myc* status, as assessed by accumulation of cells with fragmented DNA (Figure 3A) or by cell morphology (Figure 3B).

**Figure 4 pone-0008924-g004:**
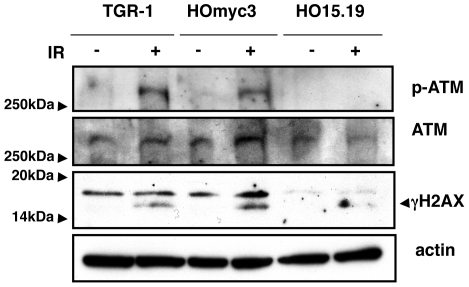
Activation of ATM and ATM-dependent responses upon induction of DNA damage is Myc dependent. TGR-1, HOmyc3, and HO15.19 cells were left untreated or exposed to IR (20Gy), and further incubated in complete medium for 2h. The levels of phospho-ATM and phospho-H2AX (γH2AX) were assessed by western blot analysis. Actin and total ATM were used as internal loading controls. One of three experiments is shown.

We next assessed whether the delayed cell death induced by irradiation in the *myc* null cells was associated with an altered ATM response. ATM phosphorylation on Ser1981 was observed in TGR-1 cells 30 min after irradiation and was sustained at least up to 8h post-treatment (Figure 4 and data not shown). In this time frame, we failed to detect any consistent ATM phosphorylation in HO15.19 cells (Figure 4 and data not shown). Reconstitution of Myc expression in HOmyc3 cell line was sufficient to rescue the ATM response (Figure 4).

ATM activation was associated with phosphorylation of the ATM substrate H2AX (γH2AX) both in the parental TGR-1 or the *myc* reconstituted HOmyc3 cells upon induction of DNA damage, while a significant reduction of γH2AX was observed in the *myc* null cells (Figure 4). Phosphorylation of the ATM substrate Chk2 could not be assessed in these cell lines, due to lack of cross-reactivity between the rat and human species of the α-phospho-CHK2 antibody.

**Figure 5 pone-0008924-g005:**
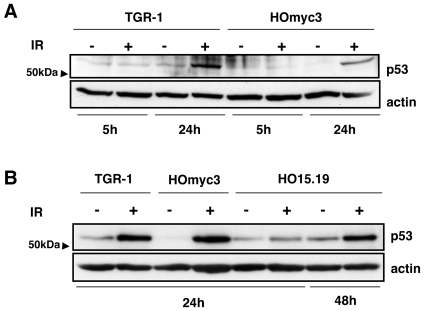
*myc* deletion prevents p53 stabilization in response to DNA damage. **A**) TGR-1 and the *myc* reconstituted HOmyc3 cells were left untreated or exposed to IR (20 Gy) for the indicated periods of time. The levels of p53 were assessed by western blot analysis. **B**) TGR-1, the *myc* reconstituted HOmyc3 and the HO15.19 cells were left untreated or exposed to IR (20 Gy) for the indicated periods of time. The levels of p53 were assessed by western blot analysis. Actin was used as an internal loading control. One out of three independent experiments is shown.

Induction of DNA DSBs by IR or CDT treatment induces ATM-dependent phosphorylation of the p53 protein on Ser15 [33], [34]. This prevents its degradation via the ubiquitin proteasome system, leading to p53 stabilization and consequent accumulation of the protein, which can be monitored by western blot analysis. Kinetics experiments showed that accumulation of p53 was a late event, detected 24h after irradiation in TGR-1 and HOmyc3 cell lines (Figure 5A). This response was further delayed in the *myc* null cells HO15.19, where stabilization of p53 was not detected until 48h after exposure to IR (Figure 5B).

**Figure 6 pone-0008924-g006:**
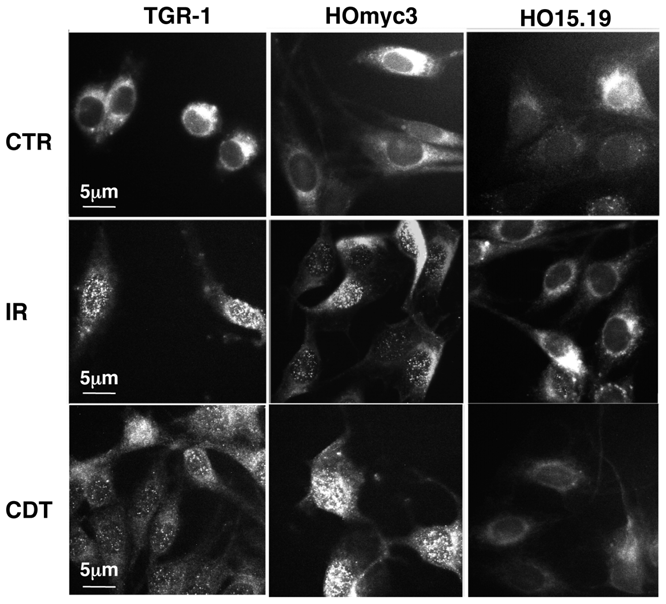
*myc* deletion impairs Nbs1 foci formation in response to DNA damage. TGR-1, the *myc* reconstituted HOmyc3, and the *myc* null HO15.19 cells were exposed to bacterial lysates (1∶2000) expressing the mutant (CTR) or the wild type form (CDT) of *H. hepaticus* CDT, or irradiated (IR, 20 Gy) and further incubated in complete medium for the indicated periods of time. The Nbs1 protein was detected by indirect immunostaining, as described in Material and Methods. The figure shows the sub-cellular distribution of Nbs1 at 2h post-treatment.

**Figure 7 pone-0008924-g007:**
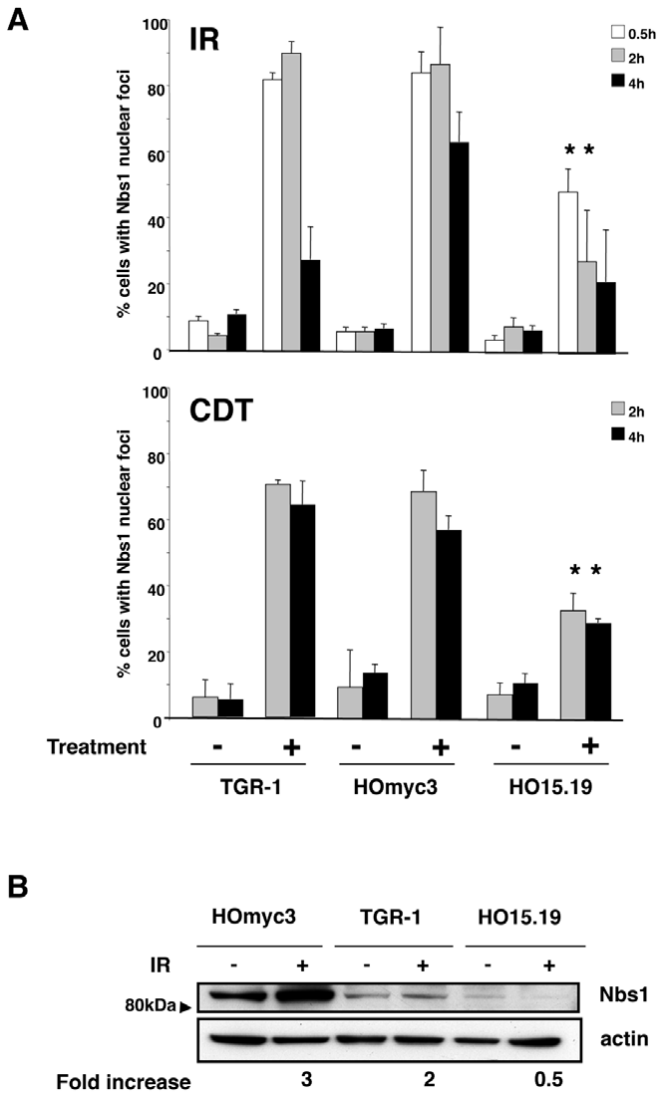
Nbs1 foci formation and Nbs1 expression is Myc dependent. **A**) Quantification of the number of cells expressing Nbs1 nuclear foci. Each bar represents the mean of three independent experiments ± SD; * statistically significant decrease in Nbs1 foci formation. A cell with more than 5 nuclear foci was scored as positive. One hundred cells were counted for each treatment. **B**) TGR-1, the *myc* reconstituted HOmyc3, and the *myc* null HO15.19 cells were left untreated or exposed to IR (20 Gy) and further incubated in complete medium for 2h. The levels of Nbs1 were assessed by western blot analysis as described in Material and Methods. The fold increased represents the ratio between the optical density of the Nbs1 specific band in irradiated cells and the optical density of the Nbs1 specific band in untreated cells. One out of three independent experiments is shown.

Efficient ATM activation is dependent on Nbs1, a member of the MRN complex, which acts as a sensor for DNA breaks [20], [21]. Therefore, we assessed the extent of Nbs1 activation in TGR-1, HOmyc3, and HO15.19 cells in response to irradiation or intoxication. As expected, a prevalent perinuclear distribution of Nbs1 was detected in the TGR-1 and HOmyc3 cells untreated or exposed to the mutant inactive CDT (Figure 6 and data not shown), while exposure to IR was associated with formation of distinct nuclear foci in 80% and 90% of the cells 30 min and 2h after irradiation, respectively (Figures 6 and 7A). The number of cells presenting Nbs1 nuclear foci started to decrease 4h after exposure to IR (Figure 7A). Similar perinuclear distribution of Nbs1 was observed in the *myc* null cells HO15.19 (Figure 6). However, irradiation-induced foci formation occurred only in approximately 40% of cells 30 min post-treatment, and the number of cells presenting nuclear Nbs1 foci further decreased at later time points (Figure 7A). A different kinetics of Nbs1 foci formation was observed in TGR-1 and HOmyc3 cells upon CDT intoxication. Up to 70% of the cells presented nuclear Nbs1 foci 2h post-intoxication, and this percentage was not significantly decreased 4h after treatment (Figure 7A). Consistent with a delayed response of the *myc* null cells to IR, nuclear re-distribution of Nbs1 was observed only in 30% to 40% of the CDT-exposed HO15.19 cells at 2h and 4h after treatment (Figures 6 and 7A).

NBS1 is described as a MYC target gene [35]. Consistent with the higher Myc levels in the HOmyc3 cells (Figure 1A), we detected enhanced expression of the Nbs1 protein in this cell line compared to the parental TGR-1, and further lower Nbs1 levels in the *myc* null cells (Figure 7B). Irradiation induced a 3-fold increase of Nbs1 expression in the HOmyc3 cells, and to a lesser extent in the TGR-1 cell line, compared to the untreated control (Figure 7B). This effect was absent in the *myc* null cells (Figure 7B).

**Figure 8:**
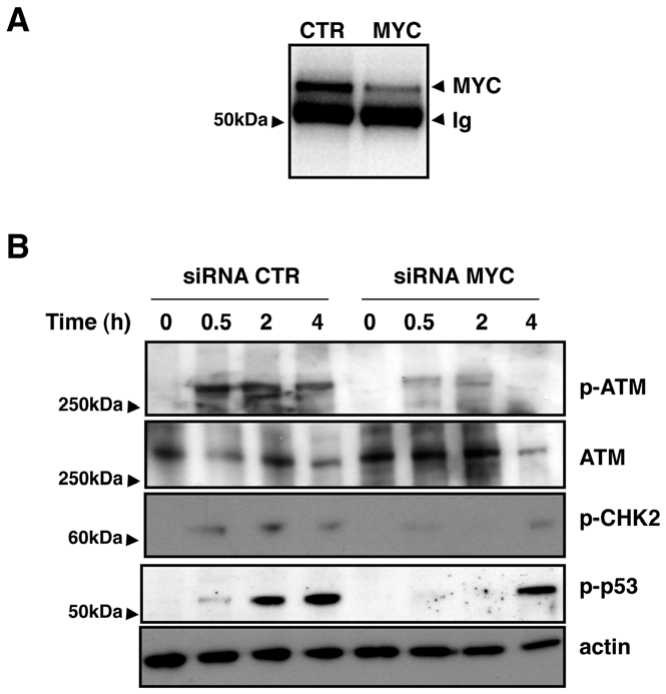
Knock down of MYC expression decreases activation of ATM, CHK2 and p53. **A**) HCT116 cells were transfected either with the control GFP-specific (CTR) or MYC specific siRNA for 48h. The expression of MYC was assessed by immunoprecipitation and western-blot analysis. One out of three independent experiments is shown. **B**) HCT116 cells, transfected either with the GFP-specific (CTR) or MYC specific siRNA were left untreated or exposed to IR (20 Gy), and further incubated in complete medium for the indicated periods of time. The levels of phosphorylation of ATM, CHK2 and p53 were assessed by western blot analysis. Actin and total ATM were used as an internal loading control. One out of three independent experiments is shown.

To confirm the contribution of MYC in the activation of the ATM dependent responses to DNA damage, we knocked down the levels of the endogenous protein in the HCT116 human cell line by siRNA. As shown in Figure 8A, a 60% decreased expression of MYC was observed in cells transfected with MYC specific siRNA compared to the levels of expression detected in cells transfected with the GFP specific siRNA (control cells). Exposure to IR induced phosphorylation of ATM on Ser1981 30 min after treatment, and the levels of phospho-ATM started to decline 4h after irradiation in control cells (Figure 8B). Similar kinetics of ATM phosphorylation was observed in the irradiated MYC depleted cells, however the levels of phospho-ATM were always significant lower. Reduced ATM activation in the MYC depleted cells was associated with decreased or delayed phosphorylation of the ATM substrates CHK2 (Thr68) and p53 (Ser15) (Figure 8B).

Collectively, these data demonstrated that MYC contributes to the regulation of the ATM-dependent checkpoint responses to DNA damage in two independent models.

## Discussion

Activation of checkpoint responses to genotoxic stress is one of the main barriers to prevent carcinogenesis (reviewed in [36]). The fate of the cell exposed to DNA damaging agents is either cell cycle arrest, eventually followed by senescence, or apoptosis (reviewed in [37]).

Physiological levels of Myc trigger apoptosis in TGR-1 cells exposed to DNA damaging agents via activation of the pro-apoptotic proteins Bax and PKC. Cell death is delayed in the *myc* null HO15.19 cells ([15], [16] and **Figure 1**). Our data demonstrate that this effect is associated with impaired activation of the ATM-dependent checkpoint responses in cells exposed to certain genotoxic agents, such as IR or CDT (**Figures 2** **to** [Fig pone-0008924-g003] [Fig pone-0008924-g004] [Fig pone-0008924-g005] [Fig pone-0008924-g006] [Fig pone-0008924-g007] **8**).

The delayed cell death in HO15.19 cells is in agreement with the late stabilization of the tumor suppressor gene p53, which was not observed until 48h post-irradiation (**Figure 5** and [38]). Delayed p53 stabilization was also observed in irradiated MYC depleted HCT116 cells compared to that observed in cells transfected with the control siRNA (**Figure 8**). While the HCT116 cells readily respond to genotoxic stress with normal induction of p53 [29] and as confirmed here, the TGR-1 cells exhibited delayed p53 stabilization, suggesting that the latter may have an altered p53 response. This is unlikely due to species differences since rapid p53 activation is induced in rat cardiac myocytes upon induction of DNA damage [39]. Our observations are in line with previous findings by Adachi et al., showing that p53 is stabilized 12h to 24h after treatment of TGR-1 cells with etoposide or cisplatin [38]. Nevertheless, Myc depletion still causes a further delay in p53 induction in these cells (**Figure 5**).

Maclean *et al* reported that MYC over-expression via a retroviral vector regulates ATM-dependent activation of p53 4h after irradiation in primary human foreskin fibroblasts [40]. However, the involvement of MYC in the regulation of upstream activators of ATM, such as NBS1, was not characterized.

Interestingly, UV irradiation elicits cell death with similar kinetics in all the three cell lines tested, independently of the *myc* status (**Figure 3**), suggesting that Myc does not contribute to cell death induced by activation of the ATR-dependent checkpoint responses [37]. These data further confirm that the delayed response to IR and CDT observed in the *myc* null cells HO15.19 was not a consequence of their slower proliferative capacity [14], [28]. The selective effect on the ATM responses may depend on the fact that NBS1, one of the proteins essential for the full kinase activation [41], is a direct target of MYC. Indeed, over-expression of MYC under a tetracycline inducible system in Epstein-Barr virus transformed B cells is associated with increased levels of NBS1 [35]. The MYC/MAX complex has also been shown to bind and enhance transcriptional activation of the *NBS1* promoter [35]. Accordingly, we observed higher levels of Nbs1 expression in the reconstituted HOmyc3 cell line, which presents approximately a 3-fold increase in Myc expression compared to the parental TGR-1 cells, while reduced levels of Nbs1 were detected in the *myc* null cells (Figures 1A and 7B). Irradiation induced enhanced Nbs1 expression in the HOmyc3 cells, and to a lesser extent in parental TGR1 cell line (Figure 7B). The pronounced increase in the Nbs1 levels in the *myc* reconstituted cells after irradiation may suggest that high Myc expression and DNA damage synergize in enhancing Nbs1 expression, which at least in part could explain why Myc overexpression sensitizes cells to DNA-damage-induced apoptosis [23].

Furthermore, we observed a strong impairment in irradiation- and intoxication-induced nuclear translocation and foci formation of Nbs1 in the HO15.19 cells (**Figures 6** **and** **7**), suggesting that Myc exerts an additional level of control, apart from gene regulation, in the DNA damage-induced activation of the MRN complex. The different kinetics of foci formation observed in irradiated versus intoxicated cells is consistent with the fact that CDT is a constantly active enzyme, which induces an increased number of lesions over time.

The delayed response of the *myc* null cells HO15.19 to CDT intoxication is quite interesting. Epidemiological evidences indicate that chronic infection with the CDT producing bacterium *Salmonella enterica serovar typhi* is associated with increased risk of hepatobiliar carcinoma [42], but the exact mechanism(s) by which bacterial infections can contribute to carcinogenesis is still poorly characterized. In light of the data reported in this work, any situation that impairs cell death in response to DNA damage induced by chronic infections with CDT-producing bacteria may promote or enhance genomic instability, and favor tumor initiation and/or progression.

**Figure 9:**
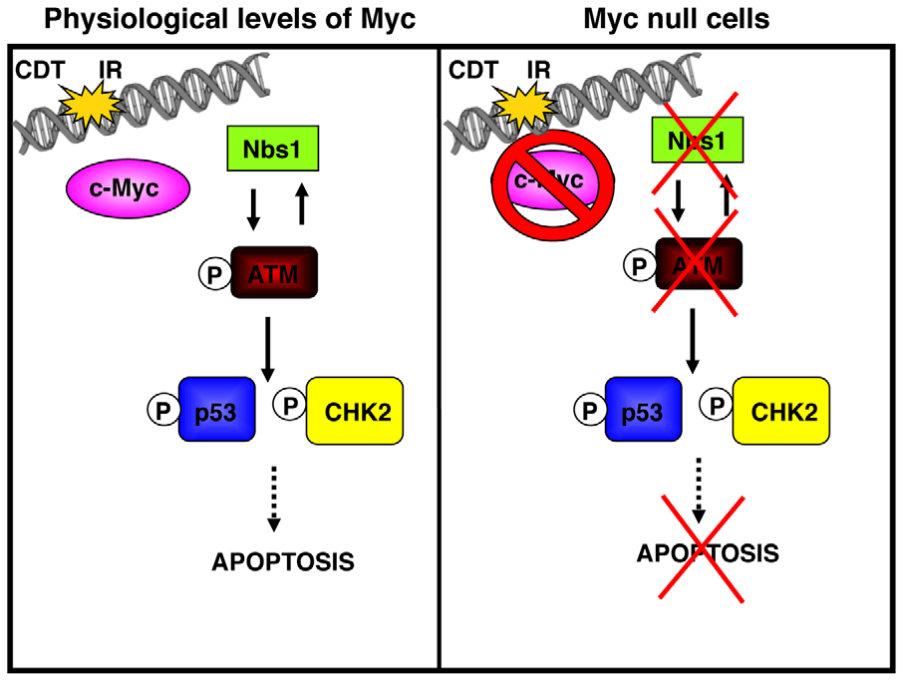
Role of MYC in the regulation of the DNA damage checkpoint responses. In response to IR or CDT, MYC contributes to activation of the ATM-dependent responses, leading to induction of apotosis. These pathways are delayed upon MYC homozygote deletion or iRNA-mediated knock down.

From the data presented in this work, some of the events regulated by MYC in response to irradiation or CDT intoxication can be summarized as follows: physiological levels of MYC are essential for the rapid nuclear foci formation of NBS1 upon DNA damage. This event triggers full activation of ATM and subsequent phosphorylation of its downstream effectors and transducer molecules, such as H2AX, CHK2 and p53 (**Figure 9**). In the presence of a substantial DNA damage that cannot be repaired, activation of the checkpoint response is likely to result in mitochondria-dependent apoptosis [15], [16], [17]. When MYC is over-expressed during cancer development, it can itself induce DNA damage both in a ROS-dependent and ROS-independent manner, thus contributing to genome instability and chromosomal abnormalities [6], [7], [8], [43].

Our results suggest that both physiological and overexpressed MYC enhances the DNA damage response by stimulating ATM phosphorylation and promoting NBS1 expression and nuclear translocation. This is consistent with the observations that inactivation of ATM diminishes MYC-induced apoptosis and augments MYC-driven tumor development [9], [40], [44]. These and our present findings have relevance for understanding the mechanisms of drug-induced cytotoxicity as well as for resistance to drug treatment in cancer therapy.
